# Report of the Committee of Distribution to the Council

**Published:** 1915-08-07

**Authors:** 


### REPORT OF THE COMMITTEE OF DISTRIBUTION TO THE COUNCIL.

The Committee of Distribution held their first meeting on Friday, May 28, and then at subsequent meetings examined the reports and income and expenditure accounts of the several institutions seeking to participate, and made their awards in conformity with the Laws of the Constitution.

The Committee beg to report that the total of the Fund on August 5 will amount to £70,600.

This year 246 institutions have applied to participate in the Fund, being seven less than in 1914, and the Committee now recommend the distribution of £69,297 to the several hospitals, institutions, dispensaries, and nursing associations shown in the appendix hereto.

The Committee having reduced the bases of award to nine institutions, the governing bodies of the same were informed thereof in accordance with the Laws of the Constitution. Six deputations attended the Committee, and, after hearing their several explanations, the bases of award were finally determined.

Seven and a half per cent. of the total sum available for distribution is appropriated to the purchase of surgical appliances during the ensuing year, and 2½ per cent. for district nursing associations.

In compliance with an order of the Council, statistics have been prepared as usual for the special use of its members, showing the number of beds in hospitals, the cost of patients at hospitals and dispensaries, the proportionate expense of management to maintenance, and other valuable information. A copy thereof has been sent to each member of the Council.

Charles Johnston, Lord Mayor, President and Treasurer.

| | |
|---|---|
| David Anderson. | G. H. Makins. |
| W. Hardy Harwood. | Adrian Pollock. |
| F. A. Bevan. | Marcus Samuel. |
| William S. Church. | C. Ernest Tritton. |
| Savile Crossley. | E. Parker Young. |

Richard B. Martin } Hon.

John Henry Buxton } Secs.

The Mansion House, July 23, 1915.

